# Supplementary figures and images for: Genome-Wide Association Study of Airway Wall Thickening in a Korean Chronic Obstructive Pulmonary Disease Cohort
Source: Genes (Basel). 2022 Jul 15;13(7):1258. doi: 10.3390/genes13071258 (PMC9318537; doi:10.3390/genes13071258)

Figure S1

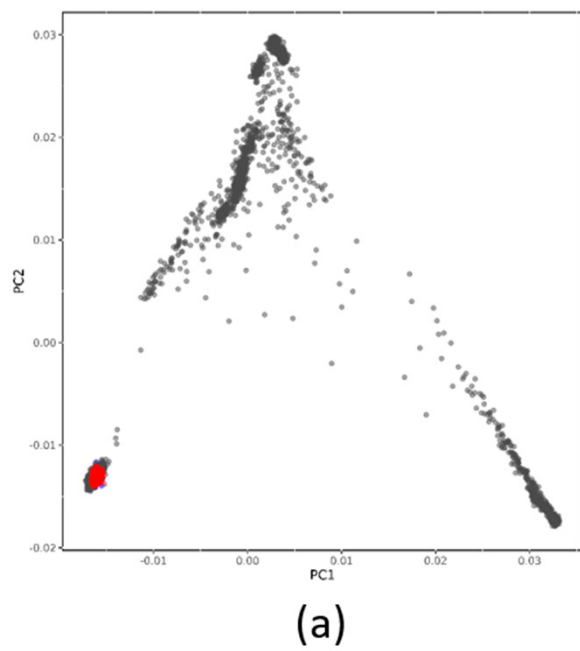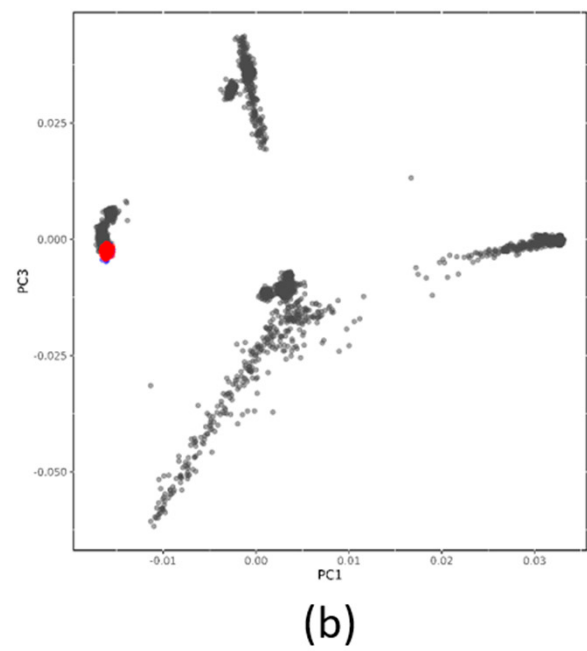

Figure S2

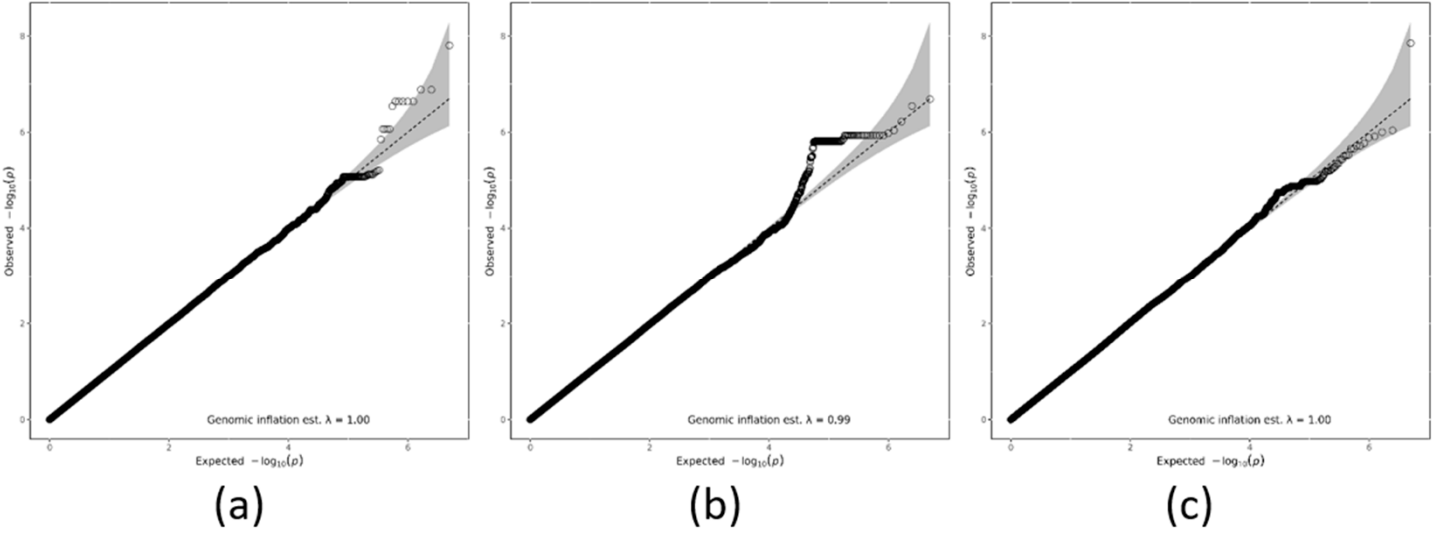

Figure S3

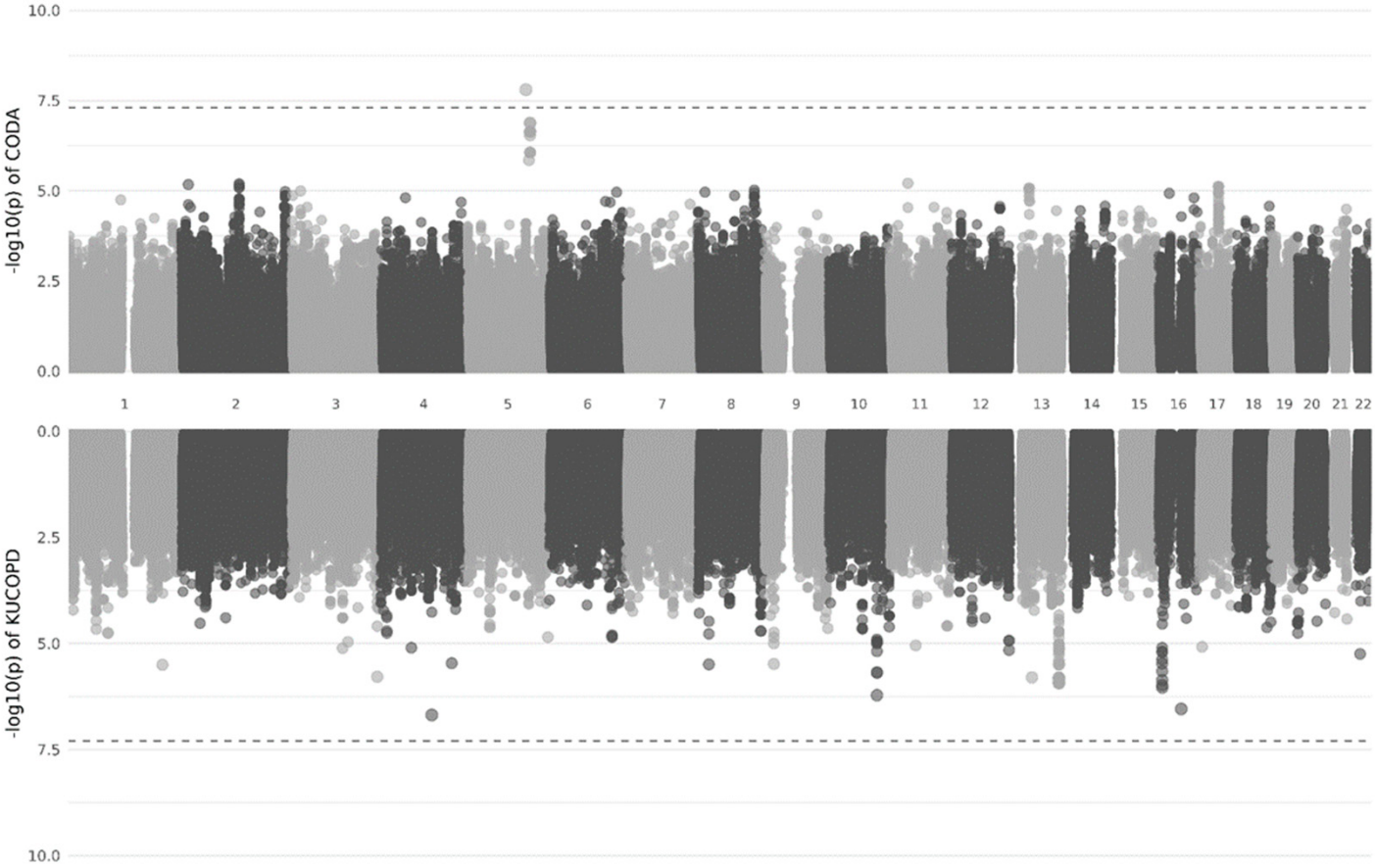

Supplement: Supplementary file 1 [file genes-13-01258-s001.zip › genes-1783265-supplementary.pdf]
